# Number of children and maternal mental health in the context of China’s fertility policy transition: the moderating effect of employment status and the mediating effect of family environment

**DOI:** 10.3389/fpsyt.2026.1780340

**Published:** 2026-04-21

**Authors:** Yuting Li, Juan Fan, Yuting Wang, Yingying Lu, Jianhua Chen, Yingyan Zhong, Qianying Hu, Shiguo Yu, Yongfen Guo, Fen Dai, Xiang Xiang, Xiaoxia Ling, Yan Wu, Yifeng Xu, Enzhao Cong

**Affiliations:** 1 Shanghai Mental Health Center, Shanghai Jiao Tong University School of Medicine, Shanghai, China; 2 China University of Petroleum-Beijing at Karamay, Karamay, China; 3 Shanghai Nanyang Middle School, Shanghai, China; 4 Attached Middle School of Xu Hui Teachers’ Institute, Shanghai, China; 5 Shanghai Tian Lin No.3 Middle School, Shanghai, China; 6 Shanghai Nanhui No.2 Middle School, Shanghai, China; 7 Shanghai Yangyuan Middle School, Shanghai, China; 8 Shanghai Tenth People’s Hospital, Tongji University School of Medicine, Shanghai, China

**Keywords:** employment status, family environment, fertility policy, maternal mental health, number of children

## Abstract

**Background:**

Having more children may be detrimental to maternal mental health during China’s ongoing fertility policy transition. However, under what circumstances and how number of children could be associated with maternal mental health remains understudied in China. This study examined the association between number of children and maternal anxiety and depressive symptoms among mothers of middle school students in Shanghai, China. It also explored the moderating effect of maternal employment status and the mediating effect of family environment.

**Methods:**

Mothers of students from 7 middle schools in Shanghai were surveyed. In total, 4,215 valid questionnaires were obtained. The survey included sociodemographic information, the Generalized Anxiety Disorder Scale (GAD-7), the Center for Epidemiologic Studies Depression Scale (CES-D), and the Chinese version of the Family Environment Scale (FES-CV). Multiple linear regression analysis was performed to examine the association between number of children and maternal anxiety/depressive symptoms. Model 1 and Model 4 of SPSS PROCESS were then employed to examine the moderating effect of employment status and the mediating effect of family environment.

**Results:**

The rates of clinically significant anxiety and depressive symptoms among mothers were 13.6% and 17.6%, respectively. The moderating effect of maternal employment status was significant. Among unemployed mothers, number of children was positively associated with both maternal anxiety and depressive symptoms, whereas among employed mothers, number of children was not associated with maternal anxiety or depression. Among unemployed mothers, family environment mediated the association between number of children and maternal anxiety/depressive symptoms through the pathways of family conflict and organization. Among employed mothers, family environment suppressed the association between number of children and maternal anxiety/depressive symptoms through the pathways of family conflict, intellectual-cultural orientation, organization, control, and independence.

**Conclusion:**

Our findings suggest that number of children per se is not necessarily associated with worsened maternal mental health. Instead, the potential changes in employment participation and family environment that accompany having more children may be more relevant. Therefore, stakeholders, clinicians, and researchers should therefore focus on these aspects when addressing maternal mental health.

## 1 Introduction

Over the past decade, China has undergone a gradual transition in fertility policy, moving from the longstanding “One-child policy” to the “Selective Two-child policy” in 2013, the “Universal Two-child policy” in 2016, and the most recent “Three-child policy” in 2021. This progressive relaxation reflects a broader shift toward encouraging larger families after decades of restrictive fertility regulations. As multi-child families become increasingly prevalent in urban China, understanding the psychological implications of raising multiple children has become essential for developing appropriate family support systems.

In China, child-rearing is characterized as a highly controlling, demanding and child-centered process that is the responsibility of mothers (1, 2). After nearly forty years of the “One-child policy”, mothers with more than one child faced the challenge of adapting to changing family structures (3). Local researchers reported higher anxiety levels in mothers of a second child compared to primiparous women (4). Studies of elderly women in China have also revealed a positive correlation between number of children and maternal depressive symptoms (5, 6). Thus, Chinese mothers may be at an increased risk of anxiety and depression when raising multiple children in the current policy environment.

However, this risk may be moderated by contextual factors such as maternal employment. Women constitute over forty percent of the Chinese labor force (7, 8). Many studies have reported a significant correlation between childbearing and a decrease in women’s labor force participation rates (9, 10) and salary levels (11, 12). While many studies have documented the bidirectional relationship between women’s fertility and employment (9–13), none have explored how these two factors can jointly affect maternal mental health. On the one hand, an increased number of children could have a more detrimental effect on employed mothers than on unemployed mothers. According to the role strain theory (14–16), employed mothers are “strained” by obligations and conflicts between their roles as mothers and employees, and increased childcare demands could result in role overload and more intense conflict. Research has found that the increasing number of children leads to increased fatigue and worsened self-rated health among female employees, students and job seekers, but not homemakers (17). The number of children was positively associated with the time pressure perceived by Australian mothers, and this effect was exacerbated among working mothers (18).

On the other hand, employment could also serve as a buffer against the adverse impact of increasing number of children. As the role enhancement theory suggests (19, 20), taking on more social roles brings benefits (e.g., greater prestige, power, security, resources, and a more consolidated identity), which tend to outweigh difficulties and increase overall well-being. Among the managerial workforce, research has found that women who committed to multiple roles report higher levels of self-esteem, self-acceptance, and life satisfaction (21). Among Chinese college students, role accumulation was found to enhance career adaptability by increasing self-efficacy and social support (22). These enhancements may offer protection with regard to child-rearing. Parents with larger social networks and greater social support have been found to experience less depression and demonstrate better psychological adjustment during the first two years of parenthood (23). Higher levels of maternal self-efficacy at three weeks after childbirth negatively predict maternal stress up to two years after childbirth and are also related to a greater decline in stress over time (24). Furthermore, evidence has shown that employed mothers generally report lower levels of depression than unemployed mothers (25, 26).

In addition to employment, the family environment is considered a critical mediator. Local scholars have noted that an increase in the number of children could lead to more complex family structures and family relationships for Chinese mothers who had experienced the “One-child policy” (3). Since the implementation of the “One-child policy”, significant changes have occurred in China in terms of childcare benefits, family parenting patterns and the ideology of motherhood. These changes include the rise of intensive parenting norms, greater investment in a single child’s future, and the emergence of the “4-2-1” family structure (four grandparents, two parents and one child), where family resources and emotional energy are heavily concentrated. Consequently, although China has a traditional concept of “many children, many blessings”, these changes have made raising multiple children an entirely new challenge. Since the implementation of the “One-child policy”, local researchers have examined differences in family environments between families with only one child and families with multiple children (27, 28), as well as the impact of family environment on children’s mental health (29, 30). However, few studies have expanded this perspective to mothers in these families.

Family environment refers to the social and environmental characteristics of a family. Studies have shown that the number of children in a family significantly influences the family environment (31–33). Boake and Salmon collected data from ten kindergartens and reported that number of children was positively related to family control and moral-religious emphasis, and negatively related to family cohesion, expressiveness and independence (31). In dual-earner families in the United States, reports by both spouses on family cohesion were negatively related to number of children (32). In British families of patients with bipolar disorder, those with more children reported higher levels of family conflict, moral-religious emphasis and control. However, the socioeconomic status did not appear to be significantly associated with the family environment (33). Due to the fertility policy, local studies examining the number of children compared only-child families with those with multiple children. Local researchers have reported that, compared with multi-child families, only-child families had higher scores for cohesion, expressiveness, independence, achievement orientation, intellectual–cultural orientation, active–recreational orientation, organization and control (27, 28). It is also worth noting that the association between number of children and family environment may vary across different cultural contexts. For example, Boake’s study found a positive relationship between number of children and family conflict (31), whereas a local study found lower levels of family control in multi-child families than in one-child families (27). This highlights the need to explore the association between number of children and family environment within China’s unique sociocultural context. Taken together, these findings suggest that family environment may serve as a mediator in the association between number of children and maternal mental health.

With the aim to better understand the relationship between number of children and maternal mental health in the context of China’s transforming fertility policy, we explored (1) whether having more children is associated with higher levels of maternal anxiety and depression; (2) whether the association is moderated by maternal employment status; and (3) whether the association is mediated by family environment. A better understanding of this association may help to identify the necessary components for early interventions in fertility-related maternal mental health issues in the context of changing family structures.

## 2 Materials and methods

### 2.1 Participants

The recruitment took place from April 23, 2021 to May 9, 2021. Mothers were recruited from seven middle schools (grades 6 to 9) in Shanghai, China. The survey was administered via an online platform by the school psychologists. At the beginning of the survey, an informed consent form was presented. The participants who selected “agree” proceeded to the survey, and those who selected “disagree” exited the survey automatically. The questionnaires were then screened: (1) by identifying IP addresses, 259 duplicate questionnaires were eliminated; (2) questionnaires with excessively long or short answer times were eliminated: answer times outside three standard deviations of the average answer time (*N* = 29) and answer times less than 2 seconds per question (*N* = 154); (3) questionnaires with outliers were eliminated (age less than 25 or greater than 100 years old, *N* = 6). Hence, a final sample of 4,215 women were included (valid response rate: 90.39%). The sample characteristics are shown in **Table 1**. This study was reviewed and approved by the Institutional Review Board of Shanghai Mental Health Center, China (Ethics Approval Number: 2021-11).

**Table 1:** Sample characteristics by number of children.

	Total sample (*N* = 4215)	Mothers of only child (*N* = 3070)	Mothers of multiple children (*N* = 1138)	
Variables	*N* (%)	*N* (%)	*N* (%)	*p*
Employment status				<0.001
Employed	3563 (84.5)	2722 (88.7)	835 (73.4)	
Unemployed	652 (15.5)	348 (11.3)	303 (26.6)	
Age [years, *M* (*P*_25_, *P*_75_)]	40 (38, 43)	40 (38, 43)	40 (38, 43)	0.890
Education ^a^				<0.001
≤ Middle school	404 (9.6)	207 (6.7)	195 (17.1)	
High/Technical	734 (17.4)	527 (17.2)	207 (18.2)	
College	2804 (66.5)	2150 (70.0)	651 (57.2)	
≥ Postgraduate	259 (6.1)	179 (5.8)	80 (7.0)	
Marital status ^a^				<0.001
Married	3915 (92.9)	2832 (92.2)	1081 (95.0)	
Remarried	47 (1.1)	25 (0.8)	22 (1.9)	
Single	26 (0.6)	22 (0.7)	4 (0.4)	
Divorced	217 (5.1)	187 (6.1)	28 (2.5)	
Family income (CNY/month)^a^				<0.001
< 5000	363 (8.6)	255 (8.3)	106 (9.3)	
5000-9999	985 (23.4)	730 (23.8)	255 (22.4)	
10000-14999	849 (20.1)	659 (21.5)	190 (16.7)	
15000-29999	1101 (26.1)	807 (26.5)	292 (25.7)	
≥ 30000	881 (20.9)	597 (19.4)	284 (25.0)	
Living status				0.006
Core family ^a^	2797 (66.4)	2039 (66.4)	756 (66.4)	
Core family and own parents	588 (14)	409 (13.3)	178 (15.6)	
Core family and husband’s parents	618 (14.7)	451 (14.7)	167 (14.7)	
Other	192 (4.6)	158 (5.1)	33 (2.9)	

^a^ Core family, living with husband and child(ren).

It is noteworthy that data collection occurred immediately prior to the announcement of the “Three-child policy” on May 31, 2021, capturing a transitional period following the implementation of the “Selective Two-child policy” (2013) and the “Universal Two-child policy” (2016). With a median age of 40 (38, 43) years, mothers in this sample likely had their second/third child after these policy changes. Although the exact timing of each births cannot be confirmed, this sample can still reflect the maternal mental health in the context of China’s fertility policy transition.

### 2.2 Measures

#### 2.2.1 Sociodemographic information

The number of children was measured by the question “How many children do you have?” Participants responded from 1 = “one child” to 3 = “three or more children” (although mothers were recruited before the implementation of the “Three-child policy”, there were situations where mothers could have three or more children, including but not limited to: they paid fines for the extra child(ren), had registered residence in rural areas or had twins). Maternal employment status was measured by the question “Are you currently employed?” The responses were dichotomous: 0 = “employed”, 1 = “unemployed”. Mothers also reported on their age, education, family monthly income, marital status and living situation.

#### 2.2.2 Maternal anxiety and depression

Anxiety was measured via the Chinese version of the 7-item Generalized Anxiety Disorder scale (GAD-7). Each item of the GAD-7 was rated on a 4-point Likert scale from 0 (not at all) to 3 (nearly every day) (34). The translated version used an optimal cutoff score of 10 (35). A score of 10 or greater was deemed as having clinically significant anxiety symptoms. Cronbach’s alpha in the current sample was 0.93. Depression was measured by the 20-item Center for Epidemiological Studies Depression Scale (CES-D). Each item was rated on a 4-point Likert scale ranging from 0 (rarely or none of the time, less than 1 day) to 3 (most or all of the time, 5–7 days) (36). The Chinese version of the CES-D has been validated for different age groups among Chinese urban population (37). A cut-off score of 16 was used in the current study. Cronbach’s alpha in the current sample was 0.96.

#### 2.2.3 Family environment

Family environment was measured via the Chinese version of the Family Environment Scale (FES-CV) (38). The scale consists of ten dimensions: cohesion (the extent to which family members are supportive, helpful, and emotionally connected to each other); expressiveness (the extent to which family members express their emotions directly and openly to each other); conflict (the extent to which family members are ambivalent, aggressive, and angry with each other); independence (the extent to which family members have a sense of independence, self-esteem, and autonomy); achievement orientation (the extent to which family members have a sense of achievement and competitiveness); intellectual-cultural orientation (the extent to which family members are interested in enhancing cultural, intellectual, political, and social activities); active-recreational orientation (the extent of family members’ participation in recreational activities); moral-religious emphasis (the extent to which family members emphasize religious, ethical, and moral values); and organization (the extent of family members’ organization and discipline in family activities) and control (the extent of family members’ demands for fixed family schedules and activities). The scale consists of 90 items measured by yes–no questions, with the responses being dichotomous: 1 = “yes”, 2 = “no”. Higher scores on each dimension indicated that the family was more likely to be characterized by that dimension. The Cronbach’s alpha coefficients for the ten dimensions in the current sample ranged from 0.54 to 0.78.

### 2.3 Statistical analysis

All statistical analyses were performed via SPSS 23.0. Among the 4,215 valid questionnaires, cases with missing values accounted for 21% of the total sample, and the missing values accounted for 2.2% of all data points. Thus, data imputation was required before the main analyses. The Expectation Maximization (EM) algorithm imputation and the Multiple Imputation (MI) methods are well evaluated and recommended by scholars (39). Since local researchers suggested that in the field of psychology, the performance of Expectation Maximization was better than that of MI (40). This study used the Expectation Maximization imputation of SPSS for missing values (except for the sociodemographic variables).

For categorical variables, descriptive information was reported as frequencies and percentages (%). The results of the Kolmogorov-Smirnov test revealed that the continuous variables were non-normally distributed, thus the median and interquartile range [*M* (*P*25, *P*75)] were used for description. The chi-square test was used to assess associations between categorical variables, the Mann-Whitney *U* test was used to assess associations between categorical and continuous variables, and the Spearman correlation coefficient was used to assess associations between continuous variables. Multiple linear regression was used to explore the relationships between number of children and symptoms of anxiety and depression while controlling for sociodemographic variables. The SPSS PROCESS model 1 was used to analyze the moderating effect of employment status and model 4 was used to analyze the mediating effect of family environment, while controlling for sociodemographic variables. When analyzing the mediating effect, first each dimension of family environment was entered separately to examine the independent mediating effect, then dimensions with a significant mediating effect were entered together into the parallel mediating model to examine the combined effect. Finally, sensitivity analyses were conducted by replicating all main analyses using complete cases from the raw dataset without the Expectation Maximization imputation.

## 3 Results

### 3.1 Sample characteristics

Among the 4,215 mothers included in the study, 3,070 (72.8%) had one child, 1,074 (25.5%) had two children, and 64 (1.5%) had three or more children. Since the number of mothers with three or more children was relatively small, they were combined with mothers of two children in the subsequent analyses, and the grouping of “number of children” was redefined as “only child = 0” and “multiple children = 1”.

The majority of mothers were employed (3,563, 84.5%). Their age ranged from 29 to 65, and the median age was 40 (38, 43) years old. In total, 3,063 (72.6%) mothers had a bachelor’s degree or above. Most of them were married (3,915, 92.9%) and had a family monthly income of more than 10,000 (2,831, 67.1%) yuan. Most of the mothers lived in their core families (2,797, 66.4%), and some lived with their own or their husbands’ parents (1,206, 28.7%). The employment rate of mothers of only child was significantly higher than that of mothers of multiple children (*χ2* = 148.43, *p* < 0.001). There were also differences in the distributions of education, marital status, monthly family income, and living status between mothers of only child and mothers of multiple children (all *p* < 0.01), as shown in **Table 1**.

### 3.2 Number of children and maternal anxiety and depression

The median score of anxiety in the current sample was 4 (1, 7), with 575 mothers (13.6%) reporting clinically significant anxiety symptoms. The median score of depression was 4 (0, 11), with 741 mothers (17.6%) reporting clinically significant depressive symptoms.

As shown in **Table 2**, number of children was not significantly associated with maternal anxiety nor depression after controlling for sociodemographic variables.

**Table 2:** Multiple regression analyses of number of children and maternal anxiety and depression.

	Anxiety symptom			Depressive symptom		
Variables	*β*	*ΔF*	*ΔR* ^2^	*β*	*ΔF*	*ΔR* ^2^
Age	-0.021			0.008		
Education	0.001			0.005		
Marital status	-0.008			0.020		
Family income	0.003			-0.041^*^		
Living status	0.046^**^			0.065^***^		
Number of children	0.001			0.007		
		(6, 4138) 1.778	0.001		(6, 4138) 5.214^***^	0.008

^*^ *p* < 0.05, ^**^p < 0.01, ^***^p < 0.001.

### 3.3 The moderating effect of employment status

**Figure 1:**
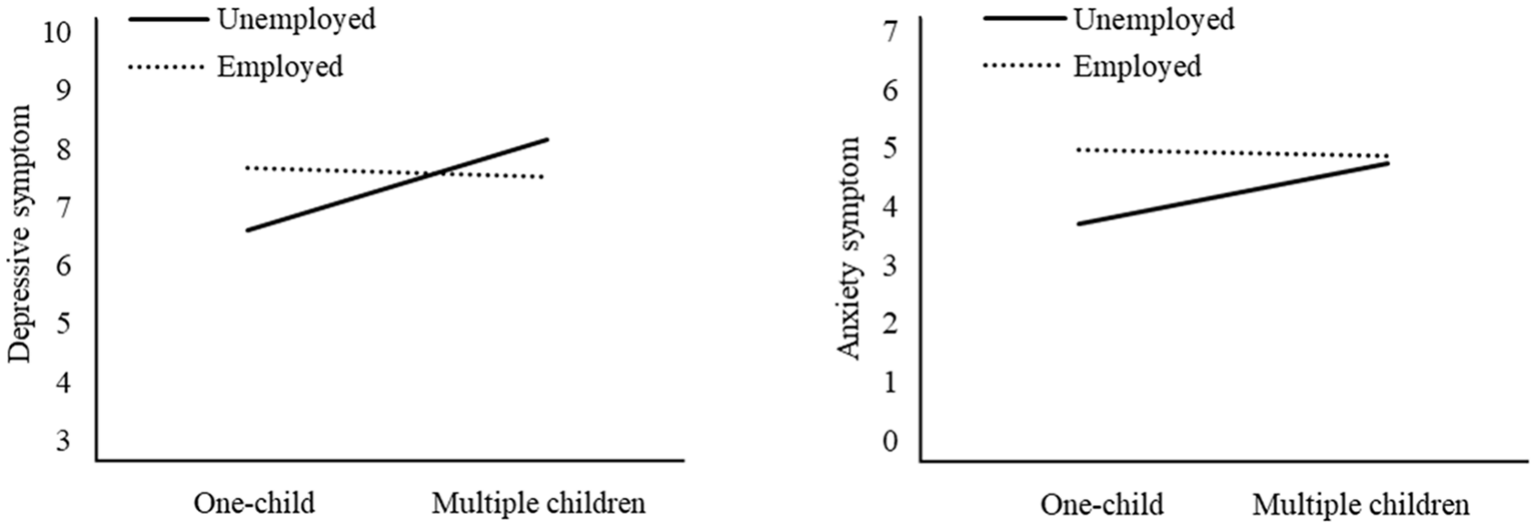
The moderation effect of maternal employment status.

As shown in **Table 3**, after controlling for sociodemographic variables, the moderating effects of maternal employment status between number of children and maternal anxiety symptoms (*β* = -0.232, *SE* = 0.089, *t* = -2.597, *p* = 0.009) and depressive symptoms (*β* = -0.177, *SE* = 0.089, *t* = -1.987, *p* = 0.047) were significant. Simple slope analyses revealed that, among unemployed mothers, number of children was positively associated with both maternal anxiety symptoms (*β* = 0.208, *SE* = 0.080, *t* = 2.604, *p* = 0.009) and depressive symptoms (*β* = 0.162, *SE* = 0.080, *t* = 2.028, *p* = 0.043). While among employed mothers, number of children was not associated with maternal mental health (see **Figure 1**).

**Table 3:** The moderation model of maternal employment status.

Variables	*β*	*SE*	*t*	*p*	95% *CI*	
Model 1: Anxiety symptom						
Constant	-0.239	0.065	-3.682	<0.001	-0.365	-0.112
Number of children	0.208	0.080	2.604	0.009	0.051	0.365
Employment status	0.261	0.059	4.452	<0.001	0.146	0.376
Number of children× Employment status	-0.232	0.089	-2.597	0.009	-0.407	-0.057
Model 2: Depressive symptom						
Constant	-0.078	0.065	-1.212	0.225	-0.205	0.048
Number of children	0.162	0.080	2.028	0.043	0.005	0.319
Employment status	0.111	0.059	1.903	0.057	-0.003	0.226
Number of children× Employment status	-0.177	0.089	-1.987	0.047	-0.352	-0.002

All analyses were controlled for socio-demographic variables.

### 3.4 The mediating effect of family environment

#### 3.4.1 Unemployed mothers

In unemployed mothers, results of the independent mediation analyses showed that family conflict (anxiety: *Effect* = 0.069, 95% *CI* = [0.013, 0.128]; depression: *Effect* = 0.065, 95% *CI* = [0.013, 0.120]) and organization (anxiety: *Effect* = 0.054, 95% *CI* = [0.013, 0.099]; depression: *Effect* = 0.027, 95% *CI* = [0.017, 0.123]) served as the independent mediators between number of children and maternal mental health (**Supplementary Table 1**), thus the two dimensions were included in the parallel mediation model.

**Figure 2:**
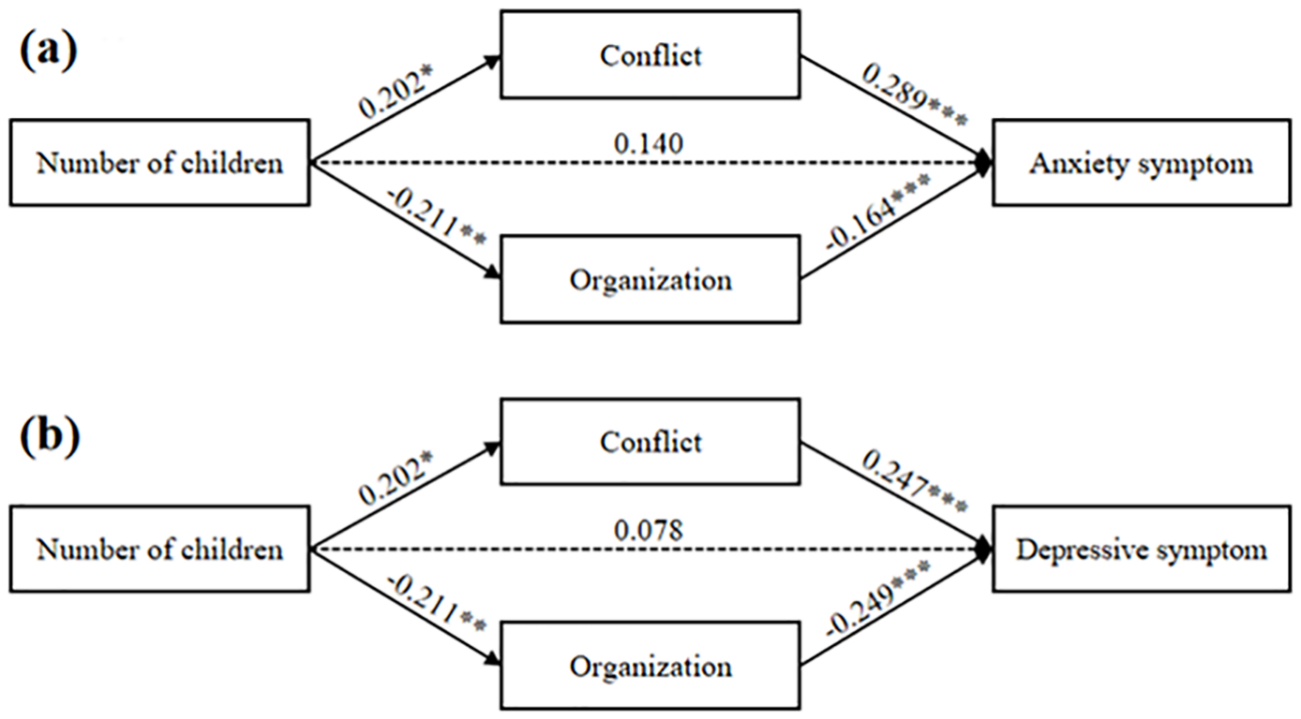
The mediation effect of family environment among unemployed mothers. ^*^p < 0.05, ^**^p < 0.01, ^***^p < 0.001.

The parallel mediation model, as shown in **Table 4**, showed that family conflict (*Effect* = 0.058, 95% *CI* = [0.012, 0.113]) and organization (*Effect* = 0.034, 95% *CI* = [0.007, 0.068]) completely mediated the association between number of children and maternal anxiety symptoms, with family conflict and organization each accounting for 25.0% and 14.7% of the total effect. As shown in **Figure 2**, number of children was positively associated with family conflict (*β* = 0.202, *SE* = 0.082, *t* = 2.474, *p* = 0.014), which was positively associated with maternal anxiety (*β* = 0.289, *SE* = 0.039, *t* = 7.478, *p* < 0.001). Number of children was negatively associated with family organization (*β* = -0.211, *SE* = 0.081, *t* = -2.612, *p* = 0.009), which was negatively associated with maternal anxiety (*β* = -0.164, *SE* = 0.039, *t* = -4.191, *p* < 0.001).

**Table 4:** The parallel mediation model of family environment among unemployed mothers.

Model path	*effect*	*SE*	95% *CI*		Mediating effect/Total effect (%)
Model 1: Anxiety symptom					
Total effect	0.232	0.081	0.074	0.391	
Number of children→ Anxiety symptom	0.140	0.075	-0.008	0.288	
Number of children→ Conflict→ Anxiety symptom	0.058	0.026	0.012	0.113	25.0
Number of children→ Organization→ Anxiety symptom	0.034	0.016	0.007	0.068	14.7
Model 2: Depressive symptom					
Total effect	0.180	0.081	0.021	0.340	
Number of children→ Depressive symptom	0.078	0.075	-0.070	0.225	
Number of children→ Conflict→ Anxiety symptom	0.050	0.022	0.009	0.097	27.8
Number of children→ Organization→ Anxiety symptom	0.053	0.022	0.013	0.099	29.4

All analyses were controlled for socio-demographic variables.

Similarly, family conflict (*Effect* = 0.050, 95% *CI* = [0.009, 0.097]) and organization (*Effect* = 0.053, 95% *CI* = [0.013, 0.099]) completely mediated the association between number of children and maternal depressive symptoms, with family conflict and organization each accounting for 27.8% and 29.4% of the total effect. Again, number of children was positively associated with family conflict, which was positively associated with maternal depressive symptoms (*β* = 0.247, *SE* = 0.038, *t* = 6.414, *p* < 0.001). Number of children was negatively associated with family organization, which was negatively associated with maternal depressive symptoms (*β* = -0.249, *SE* = 0.039, *t* = -6.420, *p* < 0.001).

#### 3.4.2 Employed mothers

Previous moderation analysis revealed that number of children was not significantly associated with maternal mental health among employed mothers. Traditionally, if the independent variable is not significantly linked to the dependent variable, mediation analysis is often deemed unnecessary (41). This premise, however, has been increasingly challenged by researchers in recent years (42, 43). A non-significant total effect does not preclude the existence of a mediation effect. For instance, the direct effect might oppose the indirect effect, or multiple mediating variables might exert counteracting influences. In such cases, a mediation or “suppression effect” may still be present even without a significant total effect. Therefore, researchers have suggested that the focus at this point should be shifted from “how the independent variable is associated with the dependent variable” to “why the independent variable is not associated with the dependent variable” (42), and the exploration of indirect effect should also be shifted from the mediation effect to the suppression effect (43). Therefore, in order to explore why number of children was not associated with maternal mental health in the current sample, the suppression effect of family environment was examined.

The results of the independent analyses showed that the indirect effects of family conflict (anxiety: *Effect* = 0.045, 95% *CI* = [0.020, 0.072]; depression: *Effect* = 0.049, 95% *CI* = [0.021, 0.079]), intellectual-cultural orientation (anxiety: *Effect* = -0.022, 95% *CI* = [-0.038, -0.007]; depression: *Effect* = -0.024, 95% *CI* = [-0.043, -0.007]), moral-religious emphasis (anxiety: *Effect* = 0.009, 95% *CI* = [0.001, 0.018]; depression: *Effect* = 0.014, 95% *CI* = [0.002, 0.028]), organization (anxiety: *Effect* = 0.024, 95% *CI* = [0.006, 0.042]; depression: *Effect* = 0.031, 95% *CI* = [0.009, 0.054]), control (anxiety: *Effect* = 0.008, 95% *CI* = [0.002, 0.015]), and independence (depression: *Effect* = -0.005, 95% *CI* = [-0.012, -0.001]) were significant (**Supplementary Table 2**), thus these dimensions were included in the parallel suppression model.

**Figure 3:**
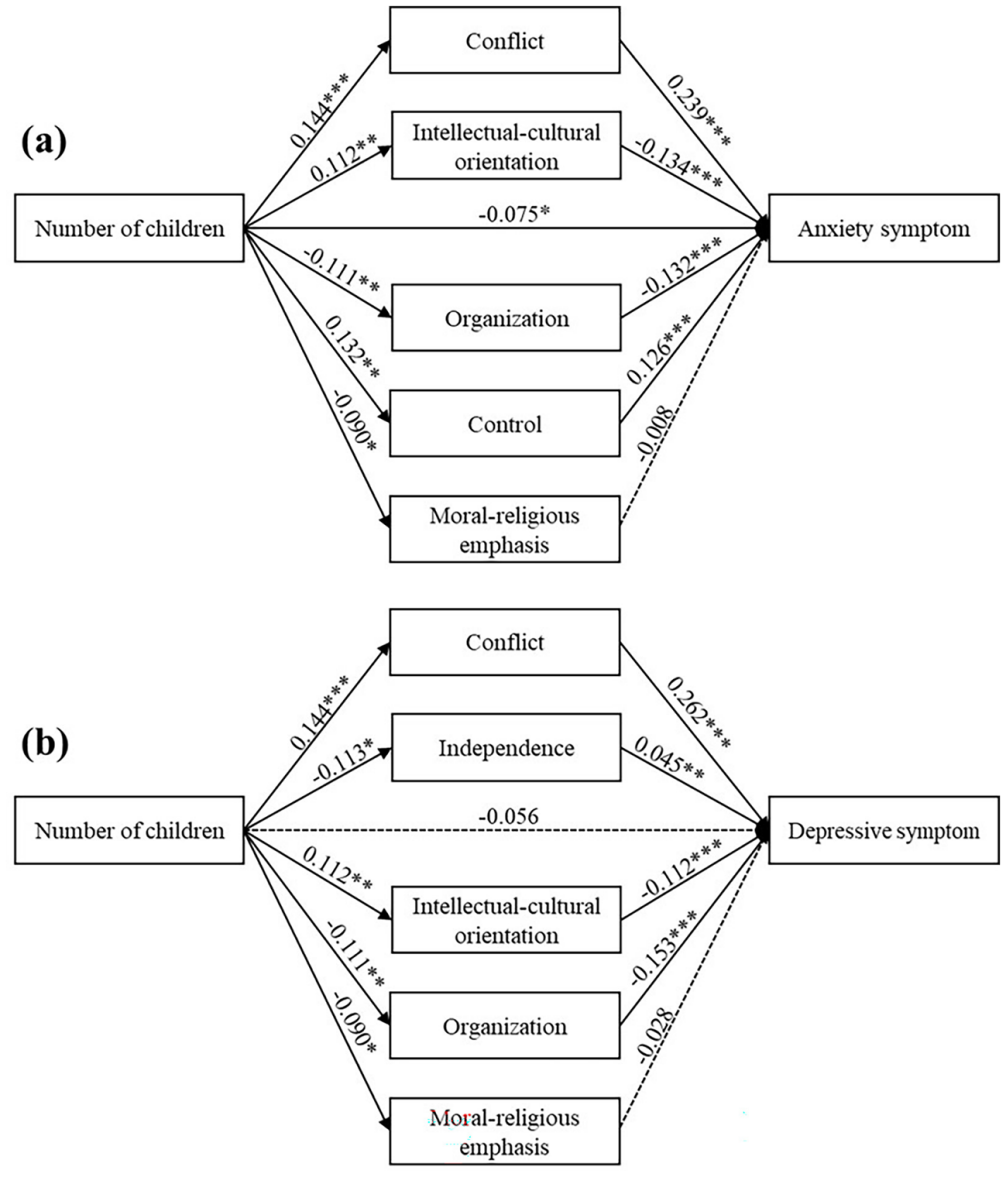
The suppression effect of family environment among employed mothers. ^*^p < 0.05, ^**^p < 0.01, ^***^p < 0.001.

In the parallel suppression model (**Table 5**), family conflict (*Effect* = 0.034, 95% *CI* = [0.015, 0.056]), intellectual-cultural orientation (*Effect* = -0.015, 95% *CI* = [-0.027, -0.005]), organization (*Effect* = 0.015, 95% *CI* = [0.004, 0.027]) and control (*Effect* = 0.017, 95% *CI* = [0.006, 0.028]) together suppressed the association between number of children and maternal anxiety symptoms. When these variables were included, the indirect effect of moral-religious emphasis turned non-significant, while the direct effect of number of children on maternal anxiety symptoms turned significant (*Effect* = -0.075, 95% *CI* = [-0.149, 0.000]). The total suppression effect accounted for 69.3% of the direct effect. Similarly, family conflict (*Effect* = 0.038, 95% *CI* = [0.017, 0.061]), independence (*Effect* = -0.005, 95% *CI* = [-0.011, -0.001]), intellectual-cultural orientation (*Effect* = -0.013, 95% *CI* = [-0.022, -0.004]) and organization (*Effect* = 0.017, 95% *CI* = [0.005, 0.031]) together suppressed the association between number of children and maternal depressive symptoms, accounting for 71.4% of the direct effect. Path coefficients are presented in **Figure 3**.

**Table 5:** The parallel suppression model of family environment among employed mothers.

Model path	*effect*	*SE*	95% *CI*		|Suppression effect/Direct effect |(%)
Model 1: Anxiety symptom					
Total effect	-0.023	0.040	-0.102	0.056	
Number of children→ Anxiety symptom	-0.075	0.038	-0.149	0.000^a^	
Number of children→ Conflict→ Anxiety symptom	0.034	0.010	0.015	0.056	45.3
Number of children→ Intellectual-cultural orientation→ Anxiety symptom	-0.015	0.006	-0.027	-0.005	20.0
Number of children→ Organization→ Anxiety symptom	0.015	0.006	0.004	0.027	20.0
Number of children→ Control → Anxiety symptom	0.017	0.005	0.006	0.028	22.7
Number of children→ Moral-religious emphasis→ Anxiety symptom	0.001	0.002	-0.003	0.005	1.3
Model 2: Depressive symptom					
Total effect	-0.017	0.040	-0.096	0.062	
Number of children→ Depressive symptom	-0.056	0.037	-0.129	0.016	
Number of children→ Conflict→ Depressive symptom	0.038	0.011	0.017	0.061	67.9
Number of children→ Independence→ Depressive symptom	-0.005	0.003	-0.011	-0.001	8.9
Number of children→ Intellectual-cultural orientation→ Depressive symptom	-0.013	0.005	-0.022	-0.004	23.2
Number of children→ Organization→ Depressive symptom	0.017	0.007	0.005	0.031	30.4
Number of children→ Moral-religious emphasis→ Depressive symptom	0.003	0.002	0.000^a^	0.007	5.4

^a^ The actual value is less than zero.

All analyses were controlled for socio-demographic variables.

### 3.5 Sensitivity analyses

To demonstrate the robustness of the findings, complete case analyses were performed using the raw dataset without data imputation. The results were largely consistent with the main analyses (**Supplementary Tables 3**-**S8**). Results of the multiple regression analyses and moderation model remained unchanged (**Supplementary Tables 3**, **S4**). In the parallel mediation models among unemployed mothers, significant mediating effects of family conflict and organization remained for both anxiety and depressive symptoms, but the direct effect of number of children on maternal anxiety symptoms became significant (**Supplementary Table 6**). Nevertheless, the reduction in effect size of the direct effect and the consistent significance of both mediators supported the central role of family environment in explaining this association. Among employed mothers, the suppression effects of family conflict, intellectual-cultural orientation, organization, and control remained significant for anxiety symptoms, while conflict, independence, and intellectual-cultural orientation remained significant for depressive symptoms (**Supplementary Table 8**). Only the suppression effect of organization became non-significant for maternal depressive symptoms. However, given that organization still exhibited a significant suppression effect on anxiety symptoms and that the overall suppression mechanism remained robust through the other pathways, this single pathway variation does not undermine the conclusion that family environment collectively buffers the association between number of children and maternal mental health among employed mothers.

## 4 Discussion

This study examined the association between number of children and maternal mental health in Shanghai, China, and explored the moderating role of employment status and the mediating role of family environment. The results showed that an increasing number of children was associated with greater anxiety and depressive symptoms in mothers, but only among the unemployed. Family environment was found to mediate the negative association between number of children and maternal mental health among unemployed mothers, while potentially suppressing the positive association between number of children and maternal mental health among employed mothers. Therefore, having multiple children per se is not necessarily associated with impaired maternal mental health, instead, it is the social and environmental characteristics of the family which change alongside increasing family size that might eventually contribute to the worsening of maternal mental health.

### 4.1 The moderation of maternal employment status

The first key finding of our study is that maternal employment status moderated the association between number of children and maternal mental health. Specifically, raising more children was only associated with higher levels of anxiety/depressive symptoms among unemployed mothers. Several previous findings have addressed the psychological burden of high parity among women globally (5, 44, 45). Although these findings did not consider maternal mental health in relation to employment status, they focused on older women who were likely to be unemployed due to retirement. The finding is in accordance with the role enhancement theory (19, 20). This theory proposes that having multiple roles helps individuals form a more consolidated identity, which is associated with improved well-being. Narrative data showed that unemployed mothers constructed their maternal identities based heavily on the behaviors and happiness of their children (46). In this sense, having more children may result in a more demanding task in terms of meeting their ideal self-image, while not being the “ideal mother” was found to be increase maternal shame and depression (47). In addition, unemployed mothers emphasized the importance of companionship (46). It is found that unemployed mothers spent considerably more time in active parenting than employed mothers (48). However, this accessibility of unemployed mothers was also accompanied by stress (46). Finally, the sociocultural context of China could also be at play. At-home mothers in China reported experiencing more stigmatization than did those in the U.S., such as being worthless to society, outdated, and ignorant (49), and having more children could have strengthened the stigma. Our findings indicate that interventions and support programs for mothers of multiple children should be tailored to address the specific challenges faced by unemployed mothers, such as enhancing social support and reducing stigma, with the goal of promoting maternal mental health and supporting fertility policies in China.

### 4.2 The mediation and suppression of family environment

The second key finding of our study was that family environment mediated/suppressed the association between number of children and maternal mental health. Specifically, among unemployed mothers, increasing number of children was associated with higher levels of family conflict and lower levels of family organization. These, in turn, were associated with higher levels of anxiety/depressive symptoms. Our results are consistent with previous findings indicating a positive association between number of children and family conflict (33). In multi-child families, parents have to devote more time and energy to raising multiple children and managing potential sibling rivalry which tends to intensify as the children grow up (50). This increased demand may exacerbate marital conflict; it has been found that number of children is negatively associated with parental marital stability (51). Additionally, the burden of parenting multiple children may also amplify parent-child conflict. Research has revealed a positive association between number of children and maternal aggressive behavior toward their children in families where mothers suffer from affective or anxiety disorders (52). Family conflict is one of the key indicators that distinguishes normal families from those affected by mental health disorders (38) and shows a significant positive correlation with parental depressive symptoms (53). Concurrently, having more children often means facing more unexpected situations, which may make it more challenging for families to maintain plans or adhere to fixed routines, thereby explaining the negative correlation between the number of children and family organization in families with middle school students (27). Previous research has shown that family organization serves as a protective factor against adolescents’ anxiety and depressive symptoms (54). This study further demonstrates that organization also acts as a protective factor against maternal anxiety and depression. Notably, once the mediating effects of family conflict and organization were accounted for, the direct effect of the number of children on maternal anxiety and depressive symptoms became non-significant. Although the concept of full mediation remains a matter of debate (42), it can be concluded that the number of children primarily induces maternal emotional symptoms by increasing adverse family environments and reducing positive ones. Moreover, among employed mothers, family conflict, organization, intellectual-cultural orientation and control collectively suppress the effect of the number of children on maternal anxiety. Specifically, conflict, organization and control exert positive indirect effects, while intellectual-cultural orientation exhibits a negative indirect effect. Similarly, the number of children increases maternal depression through the suppressing effects of family conflict and organization, whereas intellectual-cultural orientation and independence reduce maternal depression. Compared with unemployed mothers, the pathway variables linking the number of children to anxiety and depression in employed mothers additionally involve control, intellectual-cultural orientation and independence. Compared with single-child families, multi-child families exhibit stronger control, which aligns with previous international findings (31). Chinese parents tend to demonstrate high levels of control in child-rearing (1, 2), and parents with one more children may enforce discipline by upholding family rules and increasing behavioral constraints. Furthermore, parents in multi-child families may experience more parenting setbacks, prompting them to establish authority through increased psychological control (55). Contrary to domestic studies (27), mothers with multiple children report higher intellectual-cultural orientation than those with single children. From the perspective of role enhancement theory, this may reflect employed mothers’ adoption of adaptive strategies to cope with the burden of raising multiple children. Notably, this pattern emerged in a high socioeconomic status sample (72% with bachelor’s degree or higher, 67% with monthly income >10,000 CNY) drawn from affluent Shanghai districts. Within this context, fostering children’s intellectual-cultural interests through extracurricular activities, educational resources, and cultural engagement may be feasible. For instance, through fostering children’s interest in culture and knowledge and enhancing their awareness, these approaches may improve middle school students’ academic investment (56). However, this adaptive strategy may not be accessible to mothers of lower socioeconomic status. Thus, this suppressing mechanism may be context-specific, and caution is needed when generalizing to broader populations. Finally, consistent with prior domestic research (57), mothers in multi-child families report lower levels of independence. While family independence is highly valued in Western contexts, it is not necessarily viewed as favorable in Chinese families (58). The cultural emphasis on close bonds and mutual support rather than independence in Chinese families may explain why lower independence serves as a protective factor for maternal mental health. In summary, early interventions targeting the family environment in multi-child families can effectively prevent or alleviate maternal anxiety and depressive symptoms. These interventions may also transform the increase in the number of children into a positive contributor to maternal mental health, highlighting significant practical implications.

### 4.3 Strengths and limitations

As China undergoes a gradual transition in fertility policy, it is crucial to explore the impact of the number of children on maternal mental health. Currently, domestic research findings on this topic remains scarce. This study aims to contribute to this field by analyzing the relationship between the number of children and maternal anxiety and depression. It also seeks to examine the moderating role of maternal employment status. Additionally, the study explores the mechanism of how the number of children affects maternal mental health by analyzing the mediating/suppressing role of family environment. The findings have several practical implications for policymakers and practitioners. In order to support maternal mental health amidst the fertility policy transition, family-friendly public services should be strengthened, especially for mothers with multiple children. Given the moderating role of occupational status, employers and government departments should develop flexible working arrangements and mental health support programs to ease the conflict between employment and caregiving. Family environment interventions should also be integrated into public health services.

This study has several limitations. First, the sample was drawn from middle schools in the relatively affluent districts of Xuhui District and Pudong New Area of Shanghai, which may limit its representativeness. Future research should expand investigations in different areas across China. Secondly, maternal occupational status in this study was categorized into employed and unemployed, failing to account for more specific occupational circumstances, such as part-time, seeking employment, or retired. Future studies may adopt a more detailed classification of occupational status. Thirdly, although this study analyzed the effect of the number of children on maternal anxiety and depression, the cross-sectional design prevented definitive causal relationships from being reflected in the results. Moreover, self-reported measures introduces the possibility of subjective bias and reverse causality. Mothers experiencing higher levels of anxiety or depression may have reported their family environment more negatively due to negative cognitive biases. Longitudinal designs are needed to disentangle these temporal relationships. Fourthly, while the study controlled for mothers’ age, education level, marital status, living arrangements, and household income, some certain demographic variables that may influence the results were not considered, such as maternal and spousal occupational status, maternal ethnicity and household registration type. Future studies should further explore these factors. Finally, as mentioned before, the exact timing of fertility decisions relative to policy changes could not be determined. While mothers’ age suggests that mothers with multiple children likely gave birth after the policy relaxations, some may still have had additional children through multiple births or other circumstances prior to these policy changes. Future longitudinal studies with precise fertility records are needed to clarify these associations.

## 5 Conclusion

This study revealed that among unemployed mothers, having more children exacerbated their anxiety and depressive symptoms possibly due to the mediating effect of the family environment, whereas among working mothers, the family environment suppressed the effects of the number of children on maternal anxiety and depression. It is important for Chinese policymakers to address these mental health adversities while encouraging fertility. Efforts to provide social security, welfare and targeted interventions for mothers in different fertility and maternal employment situations might be especially beneficial.

## Data Availability

The raw data supporting the conclusions of this article will be made available by the authors, without undue reservation.
