# Supplementary material for: Number of children and maternal mental health in the context of China’s fertility policy transition: the moderating effect of employment status and the mediating effect of family environment
Source: Front Psychiatry. 2026 Apr 21;17:1780340. doi: 10.3389/fpsyt.2026.1780340 (PMC13139158; doi:10.3389/fpsyt.2026.1780340)
Supplement: Supplementary file 1 [file Table1.docx]

Supplementary Material

# Supplementary Figures and Tables

**Table S1.** The independent mediation models of family environment among unemployed mothers

| Variables | Anxiety symptom | | | | Depressive symptom | | | |
| --- | --- | --- | --- | --- | --- | --- | --- | --- |
|  | *effect* | *SE* | 95% *CI* | | *effect* | *SE* | 95% *CI* | |
| Cohesion | 0.007 | 0.029 | -0.050 | 0.066 | 0.010 | 0.038 | -0.065 | 0.086 |
| Expressiveness | 0.001 | 0.022 | -0.042 | 0.043 | 0.001 | 0.025 | -0.051 | 0.051 |
| Conflict | 0.069 | 0.030 | 0.013 | 0.128 | 0.065 | 0.027 | 0.013 | 0.120 |
| Independence | -0.016 | 0.011 | -0.041 | 0.003 | -0.004 | 0.012 | -0.030 | 0.020 |
| Achievement orientation | 0.002 | 0.008 | -0.014 | 0.022 | -0.001 | 0.005 | -0.012 | 0.009 |
| Intellectual-cultural orientation | 0.012 | 0.012 | -0.008 | 0.038 | 0.017 | 0.015 | -0.011 | 0.048 |
| Active-recreational orientation | 0.007 | 0.017 | -0.026 | 0.041 | 0.011 | 0.027 | -0.040 | 0.065 |
| Moral-religious emphasis | 0.002 | 0.008 | -0.012 | 0.019 | 0.013 | 0.010 | -0.001 | 0.035 |
| Organization | 0.054 | 0.022 | 0.013 | 0.099 | 0.068 | 0.027 | 0.017 | 0.123 |
| Control | 0.000 | 0.004 | -0.007 | 0.009 | -0.004 | 0.007 | -0.020 | 0.008 |
| Note. All analyses were controlled for socio-demographic variables. | | | | | | | | |

**Table S2.** The independent suppression models of family environment among employed mothers

| Variables | Anxiety symptom | | | | Depressive symptom | | | |
| --- | --- | --- | --- | --- | --- | --- | --- | --- |
|  | *effect* | *SE* | 95% *CI* | | *effect* | *SE* | 95% *CI* | |
| Cohesion | 0.004 | 0.012 | -0.020 | 0.029 | 0.005 | 0.016 | -0.026 | 0.037 |
| Expressiveness | 0.008 | 0.011 | -0.012 | 0.029 | 0.010 | 0.013 | -0.015 | 0.036 |
| Conflict | 0.045 | 0.013 | 0.020 | 0.072 | 0.049 | 0.015 | 0.021 | 0.079 |
| Independence | -0.004 | 0.003 | -0.010 | 0.000 | -0.005 | 0.003 | -0.012 | -0.001 |
| Achievement orientation | 0.002 | 0.002 | -0.001 | 0.007 | -0.001 | 0.001 | -0.004 | 0.001 |
| Intellectual-cultural orientation | -0.022 | 0.008 | -0.038 | -0.007 | -0.024 | 0.009 | -0.043 | -0.007 |
| Active-recreational orientation | -0.009 | 0.009 | -0.026 | 0.009 | -0.009 | 0.009 | -0.027 | 0.009 |
| Moral-religious emphasis | 0.009 | 0.004 | 0.001 | 0.018 | 0.014 | 0.006 | 0.002 | 0.028 |
| Organization | 0.024 | 0.009 | 0.006 | 0.042 | 0.031 | 0.012 | 0.009 | 0.054 |
| Control | 0.008 | 0.003 | 0.002 | 0.015 | 0.003 | 0.003 | -0.001 | 0.009 |
| Note. All analyses were controlled for socio-demographic variables. | | | | | | | | |

**Table S3.** Multiple regression analyses of number of children and maternal anxiety and depression using the complete case sample

|  | Anxiety symptom | | | Depressive symptom | | |
| --- | --- | --- | --- | --- | --- | --- |
| Variables | *β* | *ΔF* | *ΔR*^2^ | *β* | *ΔF* | *ΔR*^2^ |
| Age | -0.027 |  |  | 0.003 |  |  |
| Education | 0.002 |  |  | 0.011 |  |  |
| Marital status | -0.007 |  |  | 0.024 |  |  |
| Family income | 0.003 |  |  | -0.044^*^ |  |  |
| Living status | 0.050^**^ |  |  | 0.067^***^ |  |  |
| Number of children | 0.005 |  |  | 0.011 |  |  |
|  |  | (6, 4013)  2.187 | 0.003 |  | (6, 3889)  5.512^***^ | 0.008 |
| Note. ^*^*p*<0.05, ^**^*p*<0.01, ^***^*p*<0.001 | | | | | | |

**Table S4.** The moderation model of maternal employment status using the complete case sample

| Variables | *β* | *SE* | *t* | *p* | 95% *CI* | |
| --- | --- | --- | --- | --- | --- | --- |
| **Model 1: Anxiety symptom** |  |  |  |  |  |  |
| Constant | -0.244 | 0.067 | -3.659 | <0.001 | -0.374 | -0.113 |
| Number of children | 0.225 | 0.082 | 2.742 | 0.006 | 0.064 | 0.386 |
| Employment status | 0.260 | 0.060 | 4.342 | <0.001 | 0.143 | 0.377 |
| Number of children× Employment status | -0.240 | 0.091 | -2.630 | 0.009 | -0.419 | -0.061 |
| **Model 2: Depressive symptom** |  |  |  |  |  |  |
| Constant | -0.095 | 0.068 | -1.397 | 0.162 | -0.229 | 0.038 |
| Number of children | 0.197 | 0.084 | 2.356 | 0.019 | 0.033 | 0.362 |
| Employment status | 0.115 | 0.061 | 1.882 | 0.060 | -0.005 | 0.234 |
| Number of children× Employment status | -0.210 | 0.093 | -2.254 | 0.024 | -0.393 | -0.027 |
| Note. All analyses were controlled for socio-demographic variables. | | | | | | |

**Table S5.** The independent mediation models of family environment among unemployed mothers using the complete case sample

| Variables | Anxiety symptom | | | | Depressive symptom | | | |
| --- | --- | --- | --- | --- | --- | --- | --- | --- |
|  | *effect* | *SE* | 95% *CI* | | *effect* | *SE* | 95% *CI* | |
| Cohesion | 0.012 | 0.028 | -0.042 | 0.066 | 0.009 | 0.041 | -0.072 | 0.092 |
| Expressiveness | 0.006 | 0.019 | -0.032 | 0.046 | 0.002 | 0.027 | -0.051 | 0.057 |
| Conflict | 0.067 | 0.028 | 0.014 | 0.128 | 0.058 | 0.028 | 0.007 | 0.117 |
| Independence | -0.013 | 0.011 | -0.037 | 0.006 | -0.001 | 0.016 | -0.031 | 0.034 |
| Achievement orientation | 0.005 | 0.008 | -0.010 | 0.025 | -0.004 | 0.008 | -0.024 | 0.009 |
| Intellectual-cultural orientation | 0.013 | 0.011 | -0.006 | 0.039 | 0.014 | 0.017 | -0.020 | 0.050 |
| Active-recreational orientation | 0.018 | 0.016 | -0.012 | 0.054 | 0.023 | 0.030 | -0.036 | 0.085 |
| Moral-religious emphasis | 0.000 | 0.008 | -0.017 | 0.017 | 0.010 | 0.010 | -0.005 | 0.035 |
| Organization | 0.053 | 0.023 | 0.013 | 0.101 | 0.073 | 0.031 | 0.014 | 0.138 |
| Control | 0.000 | 0.004 | -0.008 | 0.010 | -0.005 | 0.010 | -0.027 | 0.013 |
| Note. All analyses were controlled for socio-demographic variables. | | | | | | | | |

**Table S6.** The parallel mediation model of family environment among unemployed mothers using the complete case sample

| Model path | *effect* | *SE* | 95% *CI* | | Mediating effect / Total effect (%) |
| --- | --- | --- | --- | --- | --- |
| **Model 1:** **Anxiety symptom** |  |  |  |  |  |
| Total effect | 0.264 | 0.081 | 0.104 | 0.425 |  |
| Number of children→ Anxiety symptom | 0.171 | 0.077 | 0.019 | 0.323 |  |
| Number of children→ Conflict→ Anxiety symptom | 0.062 | 0.025 | 0.017 | 0.115 | 23.5 |
| Number of children→ Organization→ Anxiety symptom | 0.032 | 0.016 | 0.007 | 0.069 | 12.1 |
| **Model 2: Depressive symptom** |  |  |  |  |  |
| Total effect | 0.233 | 0.089 | 0.058 | 0.408 |  |
| Number of children→ Depressive symptom | 0.137 | 0.084 | -0.027 | 0.302 |  |
| Number of children→ Conflict→ Anxiety symptom | 0.045 | 0.022 | 0.009 | 0.093 | 19.3 |
| Number of children→ Organization→ Anxiety symptom | 0.051 | 0.025 | 0.005 | 0.102 | 21.9 |
| Note. All analyses were controlled for socio-demographic variables. | | | | | |

**Table S7.** The independent suppression models of family environment among employed mothers using the complete case sample

| Variables | Anxiety symptom | | | | Depressive symptom | | | |
| --- | --- | --- | --- | --- | --- | --- | --- | --- |
|  | *effect* | *SE* | 95% *CI* | | *effect* | *SE* | 95% *CI* | |
| Cohesion | 0.004 | 0.013 | -0.021 | 0.029 | 0.004 | 0.017 | -0.028 | 0.038 |
| Expressiveness | 0.004 | 0.011 | -0.018 | 0.025 | 0.005 | 0.013 | -0.021 | 0.030 |
| Conflict | 0.043 | 0.014 | 0.017 | 0.070 | 0.043 | 0.015 | 0.015 | 0.073 |
| Independence | -0.004 | 0.003 | -0.010 | 0.000 | -0.006 | 0.003 | -0.013 | -0.001 |
| Achievement orientation | 0.002 | 0.002 | -0.001 | 0.007 | -0.001 | 0.001 | -0.005 | 0.001 |
| Intellectual-cultural orientation | -0.022 | 0.008 | -0.039 | -0.006 | -0.026 | 0.009 | -0.044 | -0.009 |
| Active-recreational orientation | -0.010 | 0.009 | -0.028 | 0.008 | -0.014 | 0.010 | -0.033 | 0.005 |
| Moral-religious emphasis | 0.008 | 0.004 | -0.000 | 0.018 | 0.012 | 0.007 | -0.001 | 0.026 |
| Organization | 0.024 | 0.009 | 0.006 | 0.042 | 0.028 | 0.012 | 0.005 | 0.053 |
| Control | 0.008 | 0.004 | 0.002 | 0.016 | 0.003 | 0.003 | -0.002 | 0.010 |
| Note. All analyses were controlled for socio-demographic variables. | | | | | | | | |

**Table S8.** The parallel suppression model of family environment among employed mothers using the complete case sample

| Model path | *effect* | *SE* | 95% *CI* | | \|Suppression effect / Direct Effect \|(%) |
| --- | --- | --- | --- | --- | --- |
| **Model 1: Anxiety symptom** |  |  |  |  |  |
| Total effect | -0.034 | 0.043 | -0.117 | 0.050 |  |
| Number of children→ Anxiety symptom | -0.080 | 0.040 | -0.159 | -0.002 |  |
| Number of children→ Conflict→ Anxiety symptom | 0.033 | 0.011 | 0.012 | 0.056 | 41.3 |
| Number of children→ Intellectual-cultural orientation→ Anxiety symptom | -0.015 | 0.006 | -0.027 | -0.005 | 18.8 |
| Number of children→ Organization→ Anxiety symptom | 0.012 | 0.006 | 0.001 | 0.025 | 15.0 |
| Number of children→ Control  → Anxiety symptom | 0.018 | 0.006 | 0.007 | 0.030 | 22.5 |
| **Model 2: Depressive symptom** |  |  |  |  |  |
| Total effect | -0.027 | 0.042 | -0.110 | 0.056 |  |
| Number of children→ Depressive symptom | -0.051 | 0.039 | -0.128 | 0.026 |  |
| Number of children→ Conflict→ Depressive symptom | 0.030 | 0.011 | 0.008 | 0.053 | 58.8 |
| Number of children→ Independence→ Depressive symptom | -0.005 | 0.003 | -0.012 | -0.001 | 9.8 |
| Number of children→ Intellectual-cultural orientation→ Depressive symptom | -0.014 | 0.005 | -0.025 | -0.004 | 27.5 |
| Number of children→ Organization→ Depressive symptom | 0.013 | 0.007 | **-0.000** | **0.027** | 25.5 |
| Note. ^a^ The actual value is less than zero.  All analyses were controlled for socio-demographic variables. | | | | | |
